# Outcomes of Patients with Anastomotic Leakage After Transhiatal, McKeown or Ivor Lewis Esophagectomy: A Nationwide Cohort Study

**DOI:** 10.1007/s00268-021-06250-w

**Published:** 2021-08-09

**Authors:** Moniek H. P. Verstegen, Annelijn E. Slaman, Bastiaan R. Klarenbeek, Mark I. van Berge Henegouwen, Suzanne S. Gisbertz, Camiel Rosman, Frans van Workum

**Affiliations:** 1 Department of Surgery and Radboud Institute of Health Sciences, Radboud University Medical Center, P.O. Box 9101, 6500 HB Nijmegen, The Netherlands; 2 Department of Surgery, Cancer Center Amsterdam, Amsterdam UMC, University of Amsterdam, Amsterdam, The Netherlands

## Abstract

**Background:**

Anastomotic leakage has a great impact on clinical outcomes after esophagectomy. It has never been studied whether anastomotic leakage is of equal severity between different types of esophagectomy (i.e., transhiatal, McKeown and Ivor Lewis) in terms of postoperative mortality and morbidity.

**Methods:**

All esophageal cancer patients with anastomotic leakage after transhiatal, McKeown or Ivor Lewis esophagectomy between 2011 and 2019 were selected from the Dutch Upper Gastrointestinal Cancer Audit (DUCA) registry. The primary outcome was 30-day/in-hospital mortality. Secondary outcomes included postoperative complications, re-operation and ICU readmission rate.

**Results:**

Data from 1030 patients with anastomotic leakage after transhiatal (*n*=287), McKeown (*n*=397) and Ivor Lewis esophagectomy (*n*=346) were evaluated. The 30-day/in-hospital mortality rate was 4.5% in patients with leakage after transhiatal esophagectomy, 8.1% after McKeown and 8.1% after Ivor Lewis esophagectomy (*P*=0.139). After correction for confounders, leakage after transhiatal resection was associated with lower mortality (OR 0.152–0.699, *P*=0.004), but mortality after McKeown and Ivor Lewis esophagectomy was similar. Re-operation rate was 24.0% after transhiatal, 40.6% after McKeown and 41.3% after Ivor Lewis esophagectomy (*P*<0.001). ICU readmission rate was 24.0% after transhiatal, 37.8% after McKeown and 43.4% after Ivor Lewis esophagectomy (*P*<0.001).

**Conclusion:**

This study in patients with anastomotic leakage confirms a strong association between severity of clinical consequences and different types of esophagectomy. It supports the hypothesis that cervical leakage is generally less severe than intrathoracic leakage. The clinical impact of anastomotic leakage should be taken into account, in addition to its incidence, when different types of esophagectomy are compared by clinicians or researchers.

## Introduction

The incidence of esophageal cancer is increasing and it is the sixth most common cause of cancer related death worldwide [1]. Approximately 30% of the patients will undergo an esophagectomy with curative intent [2, 3]. The most commonly performed procedures are transhiatal esophagectomy, (transhiatal procedure with abdominal gastric mobilization, transhiatal dissection of the lower esophagus and cervical anastomosis), McKeown esophagectomy (three stage esophagectomy with abdominal gastric mobilization, transthoracic esophageal dissection and cervical anastomosis) and Ivor Lewis esophagectomy (two stage esophagectomy with abdominal gastric mobilization, transthoracic esophageal dissection and intrathoracic anastomosis) [4]. Anastomotic leakage is a severe complication and is associated with an increased mortality rate (2–12%), a prolonged length of stay, a decreased quality of life and higher costs [5–7]. Anastomotic leakage severity can be classified according to how it was treated [8], but this does not necessarily reflect the clinical severity as can be measured by parameters like for example mortality and intensive care unit (ICU) readmission rate. Although it is often claimed that cervical anastomotic leak is less severe than intrathoracic anastomotic leak, there is little data to support this [9]. On the contrary, severe intrathoracic consequences of cervical anastomotic leakage have been described [10–13]. In addition, there might be a difference in severity of anastomotic leakage between a transthoracic and transhiatal esophagectomy with cervical anastomosis because thoracic dissection might facilitate intrathoracic manifestations of cervical anastomotic leakage (“chute hypothesis”). Although this is supported by a study including 79 patients with cervical anastomotic leakage after transthoracic versus transhiatal approach [10], patients undergoing esophagectomy with intrathoracic anastomosis were not included in this study. One study comparing mortality rates in patients with anastomotic leakage after transhiatal, McKeown and Ivor Lewis esophagectomy did not find any significant differences [14]. However, this study only included a total of 119 leakage patients and therefore anastomotic leakage severity between common types of esophagectomy remains to be studied in a large patient cohort.

The aim of this study was to investigate whether clinical outcome (severity) of anastomotic leakage was different in patients that had undergone different types of esophagectomy on a population level.

## Materials and methods

### Registry characteristics

Data were retrieved from the Dutch Upper Gastrointestinal Cancer Audit (DUCA). This is a national clinical registry that includes all surgically treated patients with esophageal, junctional or gastric cancer in the Netherlands. Participation in the DUCA is obligatory and all hospitals in the Netherlands performing esophagogastric surgery for cancer are included. Case ascertainment for the DUCA was estimated at 97.8% of all primary esophageal and junction cancer resections, as registered in the Netherlands Cancer Registry [15]. The study protocol was approved by the DUCA scientific committee and no ethical approval or informed consent was required for this study under Dutch law.

### Study cohort

Our primary aim was to investigate the severity of anastomotic leakage after different types of esophagectomy in terms of postoperative mortality and morbidity. All patients with anastomotic leakage after primary esophagectomy with gastric tube reconstruction for intrathoracic esophageal cancer or junctional cancer between 2011 and 2019 were included. General outcome data of this cohort (i.e., including patients without anastomotic leakage) have previously been described [16, 17]. In the Netherlands, different types of esophagectomy are performed, depending on the site of the lesion, patient comorbidity and surgeon preference. During the study period, esophagectomies for esophageal or junctional cancer are only performed in high volume hospitals (>20 esophagectomies per year). In this study, patients undergoing open or minimally invasive transhiatal esophagectomy or esophagectomy with 2 field lymphadenectomy (McKeown or Ivor Lewis) were included. Regarding junctional tumors, patients with a Siewert I or II tumor who underwent an esophagectomy were included in this study. Patients younger than 18 years, patients undergoing palliative or emergency resection and patients with missing data regarding the inclusion or exclusion criteria were excluded. Preoperative work-up was in accordance with local protocols and oncological staging was performed by using the TNM classification. Only patients with anastomotic leakage were selected. Anastomotic leakage was defined as full thickness gastrointestinal defect involving esophagus, anastomosis, staple line, or conduit irrespective of presentation or method of identification [8].

### Outcome parameters and definitions

The primary outcome parameter was 30-day and/or in-hospital mortality (defined as mortality from any cause during admission for esophagectomy or within 30-days after esophagectomy).

Secondary outcome parameters were pulmonary complications, cardiac complications, gastric tube necrosis (defined as a distinct outcome parameter), chyle leakage, re-intervention rate (radiologic, endoscopic or surgical) and re-operation rate (defined as for any complication during admission for esophagectomy) and ICU and hospital length of stay. In addition, a composite endpoint of re-operation and/or ICU readmission and/or 30-day/in-hospital mortality was chosen after discussions in the study team, because it reflects patients with severe clinical consequences of anastomotic leakage and not only takes into account the treatment given (as is the case in the ECCG and Clavien-Dindo classification). Since 2015, postoperative anastomotic leakage and complications were additionally scored according to the ECCG classification and Clavien-Dindo classification [8, 18].

### Statistical analysis

Patients with anastomotic leakage were selected and divided into 3 groups according the type of surgery (transhiatal, McKeown and Ivor Lewis esophagectomy) and compared for baseline and outcome parameters. To evaluate differences in baseline characteristics between the three groups, the chi-square test was used for binominal and ordinal variables. For continuous variables that did not fit a normal distribution, the Kruskal–Wallis test was used.

Binomial logistic regression analysis was performed to evaluate the effect of type of surgery on the binomial outcome parameters both with and without adjustment for potential confounding variables. Potential confounders were selected based on clinical relevance, previous literature or mechanical arguments according to recent literature advocating this method [19]. The following potential confounding variables were identified: age, gender, body mass index (BMI), American Society of Anesthesiologists (ASA) classification, neoadjuvant therapy, year of surgery and surgical approach (open or minimally invasive) [16, 20–22]. Outcomes are reported as odds ratio’s (OR’s) with a 95% confidence interval and additionally, *P* values are presented. All analyses were performed using IBM SPSS statistics software, version 25.0 (IBM Corporation, Armonk, New York, USA).

## Results

### Patient characteristics

A total of 1030 patients with anastomotic leakage after esophagectomy with gastric tube reconstruction for esophageal or junction cancer were included. Of these 1030 anastomotic leakage patients, 287 patients underwent transhiatal esophagectomy, 397 patients underwent McKeown esophagectomy and 346 patients had undergone Ivor Lewis esophagectomy. Anastomotic leakage rate was 19.7% after transhiatal esophagectomy, 16.9% after Ivor Lewis esophagectomy and 22.2% after McKeown esophagectomy. Patient, tumor and treatment characteristics according to the operation technique are shown in Table 1.

**Table 1:** Baseline characteristics

	All (*n*=1030)	Transhiatal (*n*=287)	McKeown (*n*=397)	Ivor Lewis (*n*=346)	*P* value
*Age*					
Median/IQR	66 (12)	68 (13)	65 (11)	66 (11)	<0.001
*BMI*					
Median/IQR	25.8 (5.6)	26.4 (6.1)	25.1 (5.6)	26.3 (5.6)	<0.001
*Sex*					
Male	814 (79.1%)	237 (82.6%)	287 (72.3%)	290 (83.8%)	<0.001
Female	215 (20.9%)	50 (17.4%)	110 (27.7%)	55 (15.9%)	
*ASA classification*					
1	143 (13.9%)	33 (11.5%)	67 (16.9%)	43 (12.4%)	<0.001
2	596 (57.9%)	144 (50.2%)	231 (58.2%)	221 (63.9%)	
3	276 (26.8%)	101 (35.2%)	96 (24.2%)	79 (22.8%)	
4	9 (0.9%)	5 (1.7%)	2 (0.5%)	2 (0.6%)	
Unknown	6 (0.6%)	4 (1.4%)	1 (0.3%)	1 (0.3%)	
*Tumor type*					
AC	788 (76.5%)	251 (87.5%)	240 (60.5%)	297 (85.8%)	<0.001
SCC	210 (20.4%)	26 (9.1%)	143 (36.0%)	41 (11.8%)	
Other	25 (2.4%)	8 (2.8%)	11 (2.8%)	6 (1.7%)	
Unknown	7 (0.7%)	2 (0.7%)	3 (0.8%)	2 (0.6%)	
*Tumor location*					
Proximal 1/3	17 (1.7%)	1 (0.3%)	16 (4.0%)	0 (0%)	<0.001
Middle 1/3	130 (12.6%)	9 (3.1%)	108 (27.2%)	13 (3.8%)	
Distal 1/3	658 (63.9%)	171 (59.6%)	220 (55.4%)	267 (77.2%)	
Junction	213 (20.7%)	101 (35.2%)	48 (12.1%)	64 (18.5%)	
Unknown	12 (1.2%)	5 (1.7%)	5 (1.3%)	2 (0.6%)	
*cT stage*					
T1	50 (4.9%)	20 (7.0%)	14 (3.5%)	16 (4.6%)	0.082
T2	201 (19.5%)	53 (18.5%)	74 (18.6%)	74 (21.4%)	
T3	710 (68.9%)	193 (67.2%)	276 (69.5%)	241 (69.7%)	
T4	30 (2.9%)	6 (2.1%)	18 (4.5%)	6 (1.7%)	
Unknown	39 (3.8%)	15 (5.2%)	15 (3.8%)	9 (2.6%)	
*cN stage*					
N0	358 (34.8%)	105 (36.6%)	129 (32.5%)	124 (35.8%)	0.040
N1	425 (41.3%)	105 (36.6%)	169 (42.6%)	151 (43.6%)	
N2	175 (17.0%)	54 (18.8%)	73 (18.4%)	48 (13.9%)	
N3	30 (2.9%)	4 (1.4%)	15 (3.8%)	11 (3.2%)	
N+	11 (1.1%)	3 (1.0%)	3 (0.8%)	5 (1.4%)	
Unknown	31 (3.0%)	16 (5.6%)	8 (2.0%)	7 (2.0%)	
*Neoadjuvant treatment*					
No	84 (8.2%)	39 (13.6%)	19 (4.8%)	26 (7.5%)	<0.001
Chemotherapy	45 (4.4%)	18 (6.3%)	17 (4.3%)	10 (2.9%)	
Radiotherapy	1 (0.1%)	1 (0.3%)	0 (0%)	0 (0%)	
Chemoradiotherapy	895 (86.9%)	226 (78.7%)	359 (90.4%)	310 (89.6%)	
Yes, type unknown	2 (0.2%)	1 (0.3%)	1 (0.3%)	0 (0%)	
Unknown	3 (0.3%)	2 (0.7%)	1 (0.3%)	0 (0%)	
*Year of surgery*					
2011–2012	222 (21.6%)	104 (36.2%)	92 (23.2%)	26 (7.5%)	<0.001
2013–2014	243 (23.6%)	71 (24.7%)	98 (24.7%)	74 (21.4%)	
2015–2016	280 (27.2%)	71 (24.7%)	100 (25.2%)	109 (31.5%)	
2017–2018	285 (27.7%)	41 (14.3%)	107 (27.0%)	137 (39.6%)	
*Surgical approach*					
Open	248 (24.1%)	146 (50.9%)	67 (16.9%)	35 (10.1%)	<0.001
Hybrid MIE	52 (5.0%)	0 (0%)	25 (6.3%)	27 (7.8%)	
Total MIE	730 (70.9%)	141 (49.1%)	305 (76.8%)	284 (82.1%)	

Underlined values are statistically significant (*p* < 0.05)

ASA: American society of anesthesiologists; AC: Adenocarcinoma; BMI: Body mass index; IQR: Interquartile range; MIE: Minimally invasive esophagectomy; SCC: Squamous cell carcinoma

### Primary outcome

The 30-day/in-hospital mortality rate was 4.5% (*n*=13) in patients with anastomotic leakage after transhiatal esophagectomy, 8.1% (*n*=32) in patients with anastomotic leakage after McKeown esophagectomy and 8.1% (*n*=28) in patients with anastomotic leakage after Ivor Lewis esophagectomy (*P*=0.139). When adjusting for confounding variables, mortality rate in patients with anastomotic leakage was significantly lower after transhiatal esophagectomy compared to Ivor Lewis esophagectomy (OR 0.33, 95% CI 0.15–0.70, *P*=0.004). There were no differences between McKeown and Ivor Lewis esophagectomy regarding the primary outcome (OR 0.82, 95% CI 0.46–1.45, *P*=0.49, respectively) (Table 2).

**Table 2:** Multivariate regression analysis for primary outcome parameter (30-day and/or in-hospital mortality)

	Odds ratio	95% CI	*P* value
Age	1.002	0.995–1.009	0.587
Sex	1.316	0.694–2.493	0.400
BMI	0.929	0.873–0.989	0.021
*Surgery type*			
Transhiatal	0.326	0.152–0.699	0.004
McKeown	0.817	0.461–1.448	0.489
Ivor Lewis	Ref.	Ref.	Ref.
*ASA classification*			
1	Ref.	Ref.	Ref.
2	1.599	1.171–2.584	0.309
3	3.112	1.746–4.224	0.016
4	0.000	0.000	1.000
*Year of surgery*			
2011–2012	1.279	0.560–2.923	0.559
2013–2014	1.066	0.483–2.354	0.874
2015–2016	1.895	0.963–3.730	0.064
2017–2018	Ref.	Ref.	Ref.
*Neoadjuvant therapy*			
No	1.057	0.428–2.611	0.904
Chemotherapy	0.316	0.041–2.403	0.266
Radiotherapy	0.000	0.000	1.000
Chemoradiotherapy	Ref.	Ref.	Ref.
*Surgical approach*			
Open	2.541	1.367–4.722	0.003
Hybrid MIE	1.802	0.653–4.975	0.256
Total MIE	Ref.	Ref.	Ref.

Underlined values are statistically significant (*p* < 0.05)

ASA: American society of anesthesiologists; ICU: Intensive care unit; MIE: Minimally invasive esophagectomy

### Secondary outcomes

Secondary outcome parameters are presented in Table 3. The combined endpoint of re-operation and/or ICU readmission and/or 30-day/in-hospital mortality occurred in 36.6% of anastomotic leakage patients after transhiatal esophagectomy, 55.4% after McKeown esophagectomy and 61.6% after Ivor Lewis esophagectomy (*P*<0.001). Anastomotic leakage was the reason for re-operation in 79.5% of the patients and this was chyle leakage in 2.2%, bleeding in 2.3%, “other specified” in 3% and “other, not specified” in 13.0%.

**Table 3:** Outcome parameters

	All (*n*=1030)	Transhiatal (*n*=287)	McKeown (*n*=397)	Ivor Lewis (*n*=346)	*P* value
*Mortality (30-day and/or in-hospital)*	73 (7.1%)	13 (4.5%)	32 (8.1%)	28 (8.1%)	0.139
30-day mortality	38 (3.7%)	4 (1.4%)	15 (3.8%)	19 (5.5%)	0.024
In-hospital mortality	65 (6.3%)	11 (3.8%)	31 (7.8%)	23 (6.6%)	0.103
*Pulmonary complications*	**479 (46.5%)**	**107 (37.3%)**	**181 (45.6%)**	**191 (55.2%)**	** < 0.001**
Pneumonia	89 (8.6%)	9 (3.1%)	26 (6.5%)	54 (15.6%)	< 0.001
Pleural effusion	52 (5.0%)	8 (2.8%)	14 (3.5%)	30 (8.7%)	0.001
Pneumothorax	30 (2.9%)	4 (1.4%)	14 (3.5%)	12 (3.5%)	0.197
Atelectasis	7 (0.7%)	1 (0.3%)	1 (0.3%)	5 (1.4%)	0.103
Respiratory failure	48 (4.7%)	6 (2.1%)	14 (3.5%)	28 (8.1%)	0.001
Acute aspiration	5 (0.5%)	0 (0%)	1 (0.3%)	4 (1.2%)	0.079
ARDS	13 (1.3%)	0 (0%)	7 (1.8%)	6 (1.7%)	0.079
Tracheobronchial defect	10 (1.0%)	0 (0%)	3 (0.8%)	7 (2.0%)	0.061
Persistent air leak	8 (0.8%)	0 (0%)	5 (1.3%)	3 (0.9%)	0.175
*Cardiac complications*	**244 (23.7%)**	**53 (18.5%)**	**97 (24.4%)**	**94 (27.2%)**	**0.034**
Myocardial infarction	2 (0.2%)	0 (0%)	0 (0%)	2 (0.6%)	0.138
Supraventricular arrhythmia	88 (8.5%)	10 (3.5%)	28 (7.1%)	50 (14.5%)	< 0.001
Ventricular arrhythmia	16 (1.6%)	1 (0.3%)	8 (2.0%)	7 (2.0%)	0.151
Cardiac decompensation	2 (0.2%)	0 (0%)	2 (0.5%)	0 (0%)	0.202
Pericarditis	2 (0.2%)	0 (0%)	0 (0%)	2 (0.6%)	0.138
Cardiac arrest	2 (0.2%)	0 (0%)	0 (0%)	2 (0.6%)	0.138
*Gastric tube necrosis*	**34 (3.3%)**	**6 (2.1%)**	**21 (5.3%)**	**7 (2.0%)**	**0.047**
Chyle leakage	**84 (8.2%)**	**4 (1.4%)**	**59 (14.9%)**	**21 (6.1%)**	** < 0.001**
RLN palsy	**66 (6.4%)**	**21 (7.3%)**	**41 (10.3%)**	**4 (1.2%)**	** < 0.001**
*Reintervention rate*	**688 (66.8%)**	**117 (40.8%)**	**268 (67.5%)**	**303 (87.6%)**	** < 0.001**
Radiologic*	277 (26.9%)	46 (16.0%)	108 (27.2%)	123 (35.5%)	< 0.001
Endoscopic*	394 (38.3%)	51 (17.8%)	132 (33.2%)	211 (61.0%)	< 0.001
Re-operation*	373 (36.2%)	69 (24.0%)	161 (40.6%)	143 (41.3%)	< 0.001
*ICU admission*					
LOS median days/IQR	3 (11)	2 (7)	4 (11)	5 (15)	< 0.001
ICU readmission	369 (35.8%)	69 (24.0%)	150 (37.8%)	150 (43.4%)	< 0.001
*Hospital admission*					
LOS median days/IQR	22 (24)	15 (13)	22 (25)	29 (27)	< 0.001
Hospital readmission	247 (24.3%)	67 (23.3%)	94 (23.7%)	86 (24.9%)	0.534
*Re-operation, ICU readmission and/or mortality*	**538 (52.2%)**	**105 (36.6%)**	**220 (55.4%)**	**213 (61.6%)**	** < 0.001**

Bold and underlined values are statistically significant (*p* < 0.05)

*scored as ‘yes’ relative to the total number of patients (note: one patient may underwent a radiologic reintervention and a endoscopic reintervention and a reoperation and therefore the number does not add up to 100%)

ARDS: Acute respiratory distress syndrome; ICU: Intensive care unit; IQR: interquartile range; LOS: Length of stay; RLN: Recurrent laryngeal nerve

Pulmonary complication rate was highest in patients with anastomotic leakage after Ivor Lewis esophagectomy (55.2%, *n*=191) and this was 37.3% (*n*=107) after transhiatal and 45.6% (*n*=181) after McKeown esophagectomy (*P*<0.001). Recurrent laryngeal nerve palsy was highest in patients with anastomotic leakage after McKeown esophagectomy (10.3%, *n*=41) and this was 7.3% (*n*=21) after transhiatal and 1.2% (*n*=4) after Ivor Lewis esophagectomy.

Details on the severity of anastomotic leakage according to the ECCG and Clavien-Dindo classification were available for 414 patients (Table 4). Anastomotic leakage requiring endoscopic, radiologic or surgical reintervention (ECCG grade≥2) was observed in 35.4% of the patients with anastomotic leakage after transhiatal esophagectomy, 62.0% after McKeown esophagectomy and 82.8% after Ivor Lewis esophagectomy (*P*<0.001).

**Table 4:** Anastomotic leakage according to ECCG and Clavien-Dindo classification, since registration

	All (*n*=414)	Transhiatal (*n*=65)	McKeown (*n*=163)	Ivor Lewis (*n*=186)	*P* value
*ECCG classification*					<0.001
I	135 (32.6%)	41 (63.1%)	62 (38.0%)	32 (17.2%)	
II	180 (43.6%)	18 (27.7%)	57 (35.0%)	105 (56.5%)	
III	98 (23.7%)	5 (7.7%)	44 (27.0%)	49 (26.3%)	
Unknown	1 (0.2%)	1 (1.5%)	0 (0.0%)	0 (0.0%)	
*Clavien-Dindo classification*					<0.001
I	79 (19.1%)	26 (40.0%)	45 (27.6%)	8 (4.3%)	
II	63 (15.2%)	18 (27.7%)	22 (13.5%)	23 (12.3%)	
IIIa	145 (35.0%)	8 (12.3%)	46 (28.2%)	91 (48.9%)	
IIIb	76 (18.4%)	9 (13.8%)	35 (21.5%)	32 (17.2%)	
IVa	35 (8.5%)	2 (3.1%)	12 (7.4%)	21 (11.3%)	
IVb	6 (1.4%)	2 (3.1%)	0 (0.0%)	4 (2.2%)	
V	9 (2.2%)	0 (0.0%)	3 (1.8%)	6 (3.2%)	
Unknown	1 (0.2%)	0 (0.0%)	0 (0.0%)	1 (0.5%)	

Underlined values are statistically significant (*p* < 0.05)

ECCG: Esophagectomy complications consensus group

## Discussion

This is the first nationwide study that compared the outcomes of patients with anastomotic leakage after different types of esophagectomy, investigating whether leakage after different types of esophagectomies were associated with differences in severity. After correction for confounders (among others higher age and ASA in the anastomotic leakage after transhiatal esophagectomy group), anastomotic leakage after transhiatal esophagectomy was found to be associated with a clinically relevant lower mortality rate, although this difference did not reach the level of statistical significance. In addition, anastomotic leakage after transhiatal esophagectomy was associated with the least severe consequences in terms of re-operation and ICU readmission rate, followed by McKeown and finally Ivor Lewis esophagectomy. Although a causal relationship could not be established from this type of study, these results show a strong association between severity of clinical consequences (i.e., re-operation, ICU readmission and mortality) in patients with anastomotic leakage and different types of esophagectomy. This indicates that anastomotic leakage severity should be taken into account, in addition to leakage incidence, when different types of esophagectomy are compared by clinicians or researchers. It also supports the view that a cervical anastomotic leak is often less severe than a leak from an intrathoracic anastomotic anastomosis.

In this study, transhiatal resection was performed in earlier years and it is more likely to be reserved for patients with substantial comorbidity, possibly causing selection bias. In the Netherlands, practice shifted from transhiatal toward transthoracic esophagectomy because it is believed that transthoracic esophagectomy facilitates a more extended intrathoracic lymph node dissection and therefore may result in better survival outcomes. As a result, transhiatal esophagectomy became the reserved technique for patients with significant comorbidities. However, the expected direction of the effect of this selection bias (i.e., transhiatal patients treated in earlier years and with more comorbidity are expected to do worse than patients undergoing McKeown or Ivor Lewis in later years and with less comorbidity) makes it unlikely that our study results were caused by selection bias. In fact, the difference between transhiatal and McKeown/Ivor Lewis we found may even be larger than described in the present study. On the other hand however, chemoradiotherapy was given more in later years (when surgeons more often chose McKeown or Ivor Lewis procedures) and it may be hypothesized that this precipitated more severe consequences of leakages. However, the main findings of the study persisted in multivariable analysis, in which we (among others) corrected for year of surgery and neoadjuvant treatment.

Earlier studies mainly focused on anastomotic leakage incidence and showed a significantly lower incidence of anastomotic leakage in patients with an intrathoracic as compared to a cervical anastomosis [6, 16, 23, 24]. However, our study indicates that also the severity of an anastomotic leakage is clinically relevant, since cervical anastomotic leakage was associated with less severe consequences than intrathoracic anastomotic leakage. The most used and plausible explanation is that cervical anastomotic leakage can drain through the cervical wound, preventing intrathoracic consequences of the leak, although intrathoracic manifestations can also occur [10–12]. These intrathoracic manifestations of cervical anastomotic leakage occur more frequently after McKeown than after transhiatal esophagectomy and were associated with prolonged hospital and ITU length of stay and mortality [10].

The present study also found that anastomotic leakage after McKeown esophagectomy to be more severe than anastomotic leakage after transhiatal esophagectomy which supports the clinical relevance of the “chute hypothesis”, which states that transthoracic dissection (in 2-field lymphadenectomy) can facilitate intrathoracic manifestations of cervical anastomotic leakage.

The main strength of this study is that this is a large population-based cohort study with high quality registry data which provides data on associations of outcome and type of esophagectomy in anastomotic leakage patients. Some limitations also have to be discussed. First, although the Dutch Upper GI Cancer Audit is known as a good quality and complete data registry, not all parameters of interest were available. For example, the registry does not include the reason for ICU readmission and mortality, although this information might have contributed to understanding why patients with leakage after different procedures had different outcomes regarding these parameters. However, the reason for re-operation was available in the dataset which was anastomotic leakage in 80% of the patients, suggesting anastomotic leakage was the most important contributor to the outcomes observed. Data on formal classification of anastomotic leakage according to the ECCG and Clavien-Dindo classification are only available in the DUCA registry since 2015. However, despite the smaller numbers in the sub-analyses according to the ECCG and Clavien-Dindo classification, similar results in favor of the transhiatal resection compared to the entire cohort were found. This makes it unlikely that the outcome of this study would have been different if ECCG and Clavien-Dindo would have been available for the whole study cohort and we therefore believe our results are robust. Lastly, we cannot rule out that the outcomes were affected by a learning curve. However, previous studies on learning curves in minimally invasive surgery found that the learning curve contributed to increased anastomotic leakage rate, but did not find an increased severity of anastomotic leakage (e.g., mortality rate) [25]. Also, the results of this study, adjusted for both year of surgery and surgical approach (i.e., open, hybrid or totally minimally invasive), do not point in this direction.

The fact that anastomotic leakage severity is different after different types of esophagectomy has important implications for clinicians and for future clinical research. The findings of this study should therefore be considered by clinicians who decide what type of esophageal resection they will perform for patients and this should be taken into account with other characteristics of the types of procedures (e.g., complication incidence and oncological clearance) [16, 24, 26–29]

## Conclusion

This study confirms that anastomotic leakage after different types of esophagectomy is associated with differences in outcomes and it supports the hypothesis that cervical anastomotic leakage is generally less severe than intrathoracic leakage. Leakage after transhiatal resection was associated with the lowest morbidity. For patients undergoing 2-field lymphadenectomy, a McKeown procedure was associated with lower morbidity than Ivor Lewis esophagectomy, although mortality was similar. The clinical impact of anastomotic leakage should be taken into account, in addition to its incidence, when different types of esophagectomy are compared by clinicians or researchers.
